# Blood Monocyte Chemotactic Protein-1 (MCP-1) and Adapted Disease Activity Score28-MCP-1: Favorable Indicators for Rheumatoid Arthritis Activity

**DOI:** 10.1371/journal.pone.0055346

**Published:** 2013-01-30

**Authors:** Lieh-bang Liou, Wen-pin Tsai, Chee J. Chang, Wan-ju Chao, Meng-hsin Chen

**Affiliations:** 1 Department of Rheumatology, Allergy and Immunology, Chang Gung Memorial Hospital at Lin-kou, Kwei-san Hsiang, Tao-yuan County, Taiwan; 2 Chang Gung University College of Medicine, Kwei-san Hsiang, Tao-yuan County, Taiwan; 3 Department of Rheumatology, Allergy and Immunology, Chang Gung Memorial Hospital at Kee-lung, Kee-lung City, Taiwan; 4 Clinical Informatics and Medical Statistics Research Center and Institute of Clinical Medical Science of Chang Gung University, Kwei-san Hsiang, Tao-yuan County, Taiwan; University Hospital Jena, Germany

## Abstract

**Objective:**

We assessed blood pentraxin 3 (PTX3) and macrophage chemotactic factor-1 (MCP-1) levels as indicators of disease activity in rheumatoid arthritis (RA) patients, because data on disease activity score 28 (DAS28)-erythrocyte sedimentation rate (ESR) and DAS28-C-reactive protein (CRP) are still imperfect.

**Methods:**

In 111 patients with RA, we examined longitudinal and cross-sectional correlations of blood PTX3, MCP-1, CRP, and ESR levels with measures of clinical arthritic activity, namely, swollen joint count (SJC), tender joint count (TJC), visual analog scale for general health (GH), DAS28, and adapted DAS28-MCP-1.

**Results:**

Blood MCP-1, but not PTX3, was significantly correlated with SJC, TJC, DAS28, and DAS28-CRP. DAS28-MCP-1 was strongly correlated with DAS28 (r  = 0.984, *P*<0.001) and DAS28-CRP (r  = 0.971, *P*<0.001), and modestly correlated with CRP (r  = 0.350, *P*<0.001), and ESR (r  = 0.386, *P*<0.001). Similarly, the duration of arthritic symptoms, but not sex, was significantly correlated with variables of arthritic activity. In particular, DAS28-MCP-1 significantly correlated with DAS28 during a 6-month period (r  = 0.944, *P*<0.001; r  = 0.951, *P*<0.001; r  = 0.862, *P*<0.001; and r  = 0.865, *P*<0.001 for month 0, 1, 3, and 6, respectively).

**Conclusion:**

Blood MCP-1 and adapted DAS28-MCP-1, but not blood PTX3, may be useful in monitoring RA activity.

## Introduction

Pentraxin 3 (PTX3) was first identified in human umbilical vein endothelial cells exposed to interleukin-1β (IL-1β) [1]. Other sources of PTX3 include fibroblasts, blood monocytes, neutrophilic polymorphonuclear cells (PMNs), and synovial cells [1]–[5]. To date, PTX3 is thought to have non-redundant roles in innate immunity, inflammation, matrix deposition, and female fertility [5]. The functions of PTX3 may include promoting pathogen removal, inhibiting removal of dying cells (controlling autoimmunity), and a dual role (enhancement and reduction) in complement-associated immune responses [5]. Hence, PTX3 behaves as an acute- phase protein, similar to C-reactive protein (CRP). Increased blood PTX3 levels have been associated with infection severity, prognosis of patients with acute myocardial infarctions, activation of autoreactive T cells, psoriatic skin inflammation, and severity of acute respiratory distress syndrome [6]–[10], which suggests that PTX3 mediates inflammation.

In contrast to C-reactive protein (CRP), PTX3 is produced locally by various types of cells. Furthermore, PTX3 accumulation at sites of small-vessel vasculitis [11] and PTX3-induced inhibition of phagocytosis of late apoptotic PMNs by macrophages [12] indicated that PTX3 has a key role in the leukocytoclasia in small-vessel vasculitis. High PTX3 levels have also been found in the active stages of microscopic polyangiitis, Churg-Strauss syndrome, and Wegener’s granulomatosis [13]. Moreover, elevated blood PTX3 was a marker for newly diagnosed patients with giant cell arteritis and very recent ischemia of the optic nerve [14]. Although PTX3 has been implicated in vasculitis, its role in systemic autoimmune diseases is not well understood. One study reported that unstimulated fibroblasts from patients with systemic sclerosis produced high levels of PTX3 [15]. In addition, serum PTX3 levels did not differentiate an infection group from a flare group in patients with systemic lupus erythematous (SLE) [16]. However, anti-PTX3 antibodies were more prevalent in patients with SLE than in patients with other autoimmune rheumatic diseases, which suggests that these antibodies protect SLE patients from kidney involvement [17]. There has only been one report thus far showing that patients with rheumatoid arthritis (RA) have higher levels than controls of joint fluid PTX3 [18]. RA synoviocytes, in contrast to those of osteoarthritis, constitutively displayed high PTX3 levels, without stimulation [18]. However, whether blood PTX3 level can be used as an indicator of clinical RA disease activity is unknown.

Another new mediator of inflammation, monocyte chemotactic protein-1 (MCP-1), is chemotactic for monocytes, basophils, T cells, and mast cells [19]. Activated monocytes and fibroblasts may generate MCP-1 by lipopolysaccharide (LPS) or cytokine stimulation [19]. Moreover, an MCP-1 antagonist prevented or reduced arthritis in MRL-lpr mice [20]. These studies imply that MCP-1 is significantly involved in arthritic inflammation. Nevertheless, one study unexpectedly showed that, although treatment with human monoclonal anti–CCL2/MCP-1 antibody was well tolerated by RA patients, there were no favorable immunohistologic or clinical effects [21].

The reported associations in RA patients between blood MCP-1 level and swollen joint count (SJC), and between the plasma MCP-1 level and erythrocyte sedimentation rate (ESR) and CRP level, are not entirely consistent [22], [23]. This discrepancy has not been resolved. In addition, the manner in which blood MCP-1 level relates to measures of clinical arthritis in RA patients (especially disease activity score 28: DAS28) remains unknown.

DAS28 (with inclusion of ESR) has long been used in daily clinical practice to monitor disease activity in RA patients [24]. However, DAS 28 could not accurately discriminate remission from non-remission status in RA patients [25]. Moreover, in a Japanese study, DAS28-CRP significantly underestimated RA disease activity, as compared with DAS28-ESR [26]. Therefore, it remains to be resolved whether PTX3 or MCP-1 can supplant CRP and ESR in monitoring RA disease activity by use of the DAS 28 formula and its components.

We hypothesized that RA clinical disease activity is more accurately reflected by locally produced PTX3 and MCP-1 than by CRP and ESR levels (whether produced systemically or outside the arthritic joint). To test this hypothesis, we estimated correlations between blood MCP-1, PTX3, CRP, and ESR, and SJC, tender joint count (TJC), visual analog scale for general health (GH), or disease activity score 28 (DAS28). Furthermore, we determined which of the two recently identified inflammatory mediators (PTX3 and MCP-1) was better for monitoring clinical disease activity in RA patients. Lastly, we attempted to determine whether or not DAS28-MCP-1 (see Materials and Methods) could accurately measure RA disease activity.

## Materials and Methods

### Ethics Statement

This study was conducted with the approval of the Chang Gung Institutional Review Board, Tao-yuan, Taiwan, and all subjects provided written consent for participation in the study.

### Patient Enrollment and Data Collection

We enrolled 111 consecutive patients who fulfilled the 1987 American College of Rheumatology (ACR) criteria for RA during 2006–2008. RA patients were excluded if they had infections elsewhere or acute cardiopulmonary compromise. Blood CRP, ESR, PTX3, and MCP-1 levels, and the SJC, TJC, GH, and DAS28 scores were recorded for all RA patients. The CRP level was measured by nephelometry with a lower detection limit of 3.00 mg/L; 12 of the 111 RA patients had a CRP level <3.00 mg/L and were thus assigned a CRP level of 3.00 mg/L. These data were recorded at 0, 1, 3, and 6 months for 48 newly diagnosed disease-modifying anti-rheumatic drug (DMARD)-untreated RA patients, and once for 63 RA patients who underwent standard treatment. CRP, ESR, PTX3, and MCP-1 were designated as biomarkers, whereas the SJC, TJC, GH, DAS28 [27], and DAS28-MCP-1 were designated as measures of clinical arthritic activity. DAS28-MCP-1 was calculated by incorporating MCP-1 data into the calculated equation for DAS28, as follows: DAS28-MCP-1 = 0.56×√TJC +0.28×√SJC+0.39×ln(MCP-1) +0.014× (GH). DAS28-CRP was also calculated as previously described [28].

### Antibodies for Cell Staining

Fluorescein isothiocyanate (FITC)-anti-CD66b, phycoerythrin (PE)-anti-CD11c, FITC-anti-CD62L, PE-anti-CD11b, FITC-anti-CD19, PE-anti-CD23, PE-anti-CD3, FITC-anti-CD69, and biotin-anti-MCP-1 antibodies, and streptavidin-allophycocyanin (APC) were purchased from BD Pharmingen (San Jose, CA, USA). Biotin-goat anti-human PTX3 antibody was obtained from R&D Systems (Minneapolis, MN, USA).

### Cell and Plasma Collection

Blood drawn in EDTA-containing tubes was centrifuged and separated into different cell types using the Ficoll-Hypaque technique [29]. The uppermost layer, which contains only plasma was collected, stored in a freezer at −70°C, and later tested for MCP-1 and PTX3 altogether. Monocytes and PMNs were obtained as previously described [29], [30]. B or T cells were separated by anti-CD19- or anti-CD3-magnetic beads (BD, Mountain View, CA, USA). The purity of separated cells was >95% and all cells were suitable for staining.

### Measurements of Plasma PTX3, MCP-1, and Anti-cyclic Citrullinated Peptide (CCP) Antibody Levels

ELISA assays for PTX3 or MCP-1 were done on plasma according to the manufacturer’s recommendations (R&D Systems). The intra-assay and inter-assay coefficient of variance (CVs) for the PTX3 measurement were <4% and <10%, respectively. The intra-assay and inter-assay CVs for the MCP-1 measurement was <5% and <9%, respectively. Because plasma PTX3 or MCP-1 data were collected after all clinical examinations of arthritic activity were completed, we excluded possible confounding effects in the detection of correlations between plasma PTX3 and MCP-1 levels and measures of arthritic activity. The anti-CCP antibodies in plasma were quantified using Quanta Lite CCP3 IgG ELISA (Inova Diagnostics, Inc., San Diego, CA, USA): the intra-assay and inter-assay C.V. for anti-CCP measurement was **<**7% or **<**8%, respectively.

### Flow Cytometric Analysis of Intracellular PTX3 and MCP-1 and Cell Surface Marker Staining in Activated Peripheral Blood Leukocytes

Blood cells obtained as described above were stained with individual agents on the cell surface at 4°C for 30 minutes. After being washed twice with phosphate- buffered saline (PBS), cells were mixed with 250 µl of BD fixation/permeabilization solution and maintained at 4°C for 20 minutes. Then, 100 µl of BD Perm/Wash solution was added to the cells, which were again maintained at 4°C for 30 minutes. Then, biotin-anti-MCP-1 and biotin-anti-PTX3 antibody were mixed with cells at 4°C for 30 minutes. Finally, streptavidin-APC was used lastly to stained cells at 4°C for 30 minutes. After again twice washing with PBS, cells were run on a BD flow cytometer for fluorescence detection. Cell surface L-selectin (CD62L) and CD11b [31] were used to identify activated monocytes. Activated PMNs were designated as CD11c^+^CD66b^+^. In summary, individual cell types were stained with appropriate FITC- or PE-coated antibodies for cell surface markers, and were stained intracellularly with biotin-anti-PTX3 or biotin-anti-MCP-1 antibodies followed by streptavidin-APC.

### Statistical Analysis

The SPSS 16.0 software package was used for data analysis. One-sample Kolmogorov-Smirmov Z test was used to test the data normality. Correlations between sex, duration of arthritic symptoms, plasma PTX3 and MCP-1 levels, blood CRP and ESR levels (the independent variables), and RA arthritic activity (SJC, TJC, DAS28, DAS28-MCP-1, and DAS-CRP; all outcome dependent variables except GH were normally distributed) were detected by Pearson’s correlation coefficients (shown as r-values). Spearman’s correlation (shown as rho-values) was used to estimate correlations with the outcome dependent variable GH, which was not normally distributed. Correlations were also estimated after stratification of CRP and ESR levels. Multiple linear regression analysis of measures of clinical arthritic activity (except GH which was not normally distributed) and sex, duration of arthritic symptoms, MCP-1, PTX3, CRP, and ESR levels was performed by multivariate analysis of variance (ANOVA). Correlations between intracellular staining levels of activated cells and plasma PTX3 or MCP-1 levels and measures of clinical arthritic activity were determined by Spearman’s correlation coefficient (shown by rho-values). The comparison of different data groups that were not normally distributed was analyzed by Mann-Whitney U test. A *P* value of <0.05 was considered statistically significant. Because newly-diagnosed RA patients were less likely to be diagnosed at our tertiary care center (and were followed for a 6-month period), a *P* value ≥0.05 and <0.10 was defined as weakly significant [32].

## Results

### Clinical and Laboratory Characteristics of Enrolled RA Patients

The laboratory and clinical data of the 111 RA patients are shown in Table 1. The mean (±SD) blood PTX3 level in the 41 age- and sex-matched healthy controls was 1.48±0.23 ng/ml (range 0.00–8.37), which is similar to that in a previous report [33]. The blood MCP-1 level in our healthy controls was 70.25±16.70 pg/ml (range 0.00–473.34), which is also similar to that in a previous report [34]. The median plasma MCP-1 level of the RA patients (Table 1) was significantly higher than that of the healthy controls (median 0.00 pg/ml; 25^th^ and 75^th^ percentiles, 0.00 and 120.21, respectively; *P*<0.001). However, the difference in median PTX3 was not significant (median, 0. 98 ng/ml; 25th and 75th percentiles, 0.55 and 1.56, respectively; *P*  = 0.315). The 48 newly diagnosed RA patients were followed for >1 year (several were contacted by telephone). At this writing, no patient has developed another autoimmune disorder.

**Table 1:** Demographic, laboratory, and clinical characteristics of enrolled RA* patients.

	Mean [SD]	Range
Number of patients	111	
Age, years	51.4 [12.1]	17–78
Gender (female/male)	89/22	
Duration of arthritic symptoms, months+	24.0 [4.0, 68.0]	1.5–207.0
RF-positive rate	59%	
Anti-CCP-positive rate	61%	
MCP-1, pg/mL+	58.63 [31.5, 160.35]	10.72–1090.16
PTX 3, ng/mL+	0.92 [0.49, 1.55]	0.01–58.23
CRP, mg/L+	10.10 [3.30, 28.60]	0.75–129.00
ESR, mm/hr	41.29 [28.56]	4–128
Swollen joint count	5.18 [4.57]	0–22
Tender joint count	6.68 [5.46]	0–28
GH+	100 [25, 100]	0–100
DAS28	5.19 [1.60]	1.57–8.57
DAS28-MCP-1	4.87 [1.55]	2.37–9.86
DAS28-CRP	4.66 [1.55]	1.48–7.91

* RA, rheumatoid arthritis; Mean [SD], mean ± standard deviation; RF, IgM rheumatoid factor; Anti-CCP, anti-cyclic citrullinated peptide 3 antibody; MCP-1, macrophage chemotactic protein-1; PTX3, pentraxin 3; CRP, C-reactive protein; ESR, erythrocyte sedimentation rate; GH, visual analog scale for general health [maximum score  = 100]; DAS28, disease activity score 28 [ESR included]; DAS28-MCP-1, disease activity score 28 [MCP-1 included instead of ESR]; DAS28-CRP, disease activity score 28 [CRP included]. +Data shown are median [25th percentile, 75th percentile]. Serum rheumatoid factors were examined at diagnosis (newly diagnosed RA patients) and were not subsequently re-checked, however, 33% of regularly treated RA patients had RF re-checked on enrollment due to loss of the data; anti-CCP antibodies were measured on enrollment for all RA patients.

### Correlation between Laboratory and Clinical Variables

Plasma MCP-1 level was modestly but significantly correlated (r <0.500) with DAS28 in the 111 RA patients (Table 2). Similarly, serum CRP and blood ESR were modestly but significantly correlated (r <0.500) with DAS28 (Table 2). The correlations of CRP and ESR with DAS28 were similar (Table 2). In contrast, plasma PTX3 was not correlated with DAS28 (Table 2). Intriguingly, plasma MCP-1 was correlated with two components of DAS28 (SJC and TJC) excluding the laboratory marker. In contrast, CRP and ESR were correlated with only one component (SJC) (Table 2). Duration of arthritic symptoms, but not sex, was significantly correlated with all indicators of arthritic activity (Table 2).

**Table 2:** Univariate analysis of correlations between independent markers and measures of clinical arthritic activity.

	SJC	TJC	GH*	DAS28	DAS28-MCP1	DAS28-CRP
Sex	−0.054 (0.570)	−0.129 (0.177)	−0.090 (0.346)	−0.105 (0.271)	−0.083 (0.389)	−0.057 (0.435)
Duration of symptoms	−0.400 (<0.001)	−0.418 (<0.001)	−0.467 (<0.001)	−0.432 (<0.001)	−0.440 (<0.001)	−0.419 (<0.001)
MCP-1	0.312 (0.001)	0.233 (0.014)	0.149 (0.119)	0.307 (0.001)	0.406 (<0.001)	0.313 (0.001)
PTX3	0.015 (0.877)	0.009 (0.928)	−0.073 (0.444)	0.157 (0.100)	0.146 (0.127)	0.141 (0.140)
CRP	0.245 (0.010)	0.113 (0.239)	0.250 (0.008)	0.407 (<0.001)	0.350 (<0.001)	0.435 (<0.001)
ESR	0.276 (0.003)	0.146 (0.127)	0.207 (0.029)	0.493 (<0.001)	0.386 (<0.001)	0.352 (<0.001)

* Spearman’s correlation. All other correlations (r) are Pearson’s correlation coefficients for 111 patients with rheumatoid arthritis and r (*P*) values are shown. MCP-1, macrophage chemotactic factor-1; PTX3, pentraxin 3; CRP, C-reactive protein; ESR, erythrocyte sedimentation rate; SJC, swollen joint count; TJC, tender joint count; GH, visual analog scale for general health; DAS28, disease activity score 28 [ESR included]; DAS28-MCP-1, disease activity score 28 [MCP-1 included instead of ESR]; DAS28-CRP, disease activity score 28 [CRP included].

**Figure 1:**
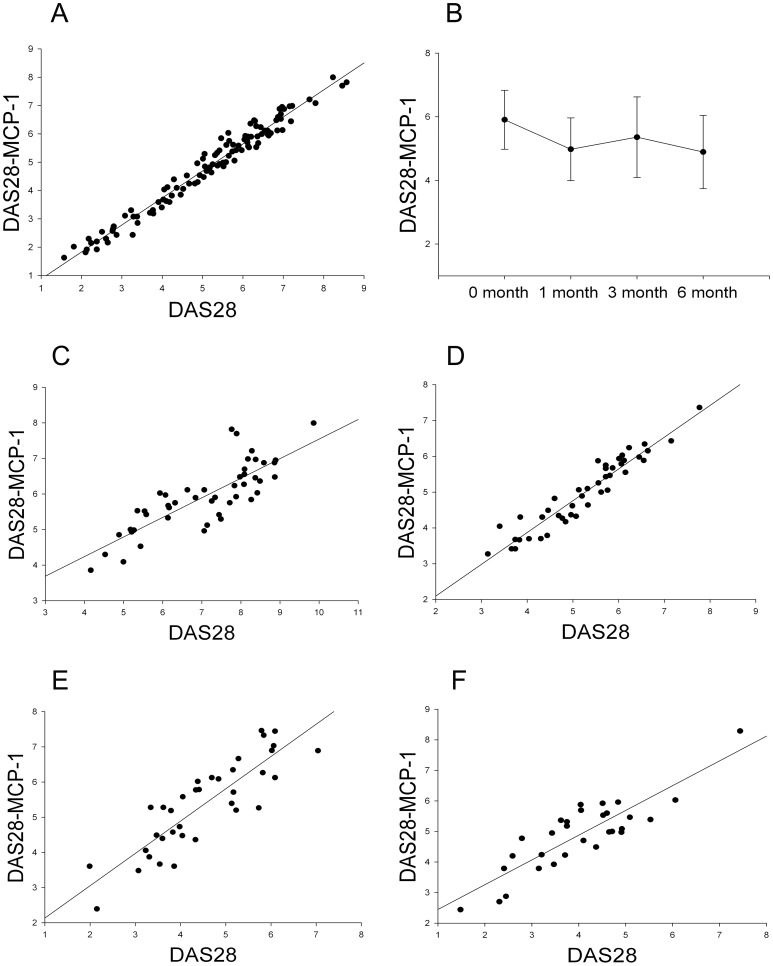
Performance and correlation of DAS28-MCP-1 with DAS28. Correlation analysis was performed by estimating Pearson’s correlation coefficients for 111 patients with rheumatoid arthritis, including 48 newly diagnosed (blood drawn four times in a 6-month period) patients and 63 patients undergoing standard treatment (blood drawn only once). (A) Overall correlation between DAS28-MCP-1 and DAS28 (48 newly diagnosed patients at 0 months plus 63 patients) (r  = 0.984 and *P*<0.001). (B) Change in DAS28-MCP-1 during a 6-month period was assessed by ANOVA and *post-hoc* LSD test. The *P*-values were <0.001, 0.022, and <0.001 for the comparison of 0 (n  = 48) months, with 1 (n  = 44), 3 (n  = 36), and 6 months (n  = 30), respectively. Values were expressed as mean ± SD. (C) Correlation between DAS28 and DAS28-MCP-1 at 0 months among newly diagnosed patients (r  = 0.944 and *P*<0.001). (D) Correlation between DAS28 and DAS28-MCP-1 at 1 months among newly diagnosed patients (r  = 0.951 and *P*<0.001). (E) Correlation between DAS28 and DAS28-MCP-1 at 3 months among newly diagnosed patients (r  = 0.862 and *P*<0.001). (F) Correlation between DAS28 and DAS28-MCP-1 at 6 months among newly diagnosed patients (r  = 0.865 and *P*<0.001).

Interestingly, the correlation between DAS28-MCP-1 score and DAS28 was strong (111 RA patients, Figure 1A), and slightly better than the correlation of r  = 0.961 between DAS28-CRP and DAS28. DAS28-MCP-1 score decreased significantly during the 6-month treatment period (Figure 1B). Moreover, DAS28-MCP-1 score correlated highly with DAS28 at 0, 1, 3, and 6 months of the 48 newly diagnosed RA patients (Figures. 1C–1F). DAS28-MCP-1 score in the 111 RA patients was modestly but significantly correlated with the CRP and ESR (Table 2). Moreover, DAS28-MCP-1 score correlated highly with DAS28-CRP (r  = 0.971; *P*<0.001). These results suggest that the adapted DAS28-MCP-1 is a useful indicator of clinical disease activity in RA.

Interestingly, multivariate analysis of the 111 RA patients revealed that plasma MCP-1 level accurately predicted clinical arthritic activity and was more useful than ESR level in predicting such activity (especially for SJC and TJC) (Table 3). Similarly, duration of arthritic symptoms, but not sex, was significantly correlated with all measures of arthritic activity (Table 3).

**Table 3:** Multivariate analysis of correlations between independent markers and measures of clinical arthritic activity.

	SJC	TJC	DAS28	DAS28-MCP1	DAS28-CRP
Sex	0.474 [(−2.738) −1.282]	0.108 [(−4.495) −0.453]	0.087 [(−1.174) −0.081]	0.087 [(−1.156) −0.080]	0.115 [(−1.143) −0.125]
Duration of symptoms	<0.001 [(−0.049)– (−0.017)]	<0.001 [(−0.065)– (−0.024)]	<0.001 [(−0.017)– (−0.006)]	<0.001 [(−0.016)– (−0.006)]	<0.001 [(−0.017)– (−0.006)]
MCP-1	0.006 (0.001 to 0.009)	0.035 (0.000 to 0.009)	0.009 (0.000 to 0.003)	<0.001 (0.001 to 0.003)	0.021 (0.000 to 0.003)
PTX3	0.235 [(−0.182) −0.045]	0.695 [(−0.168) −0.112]	0.696 [(−0.035) −0.036]	0.907 [(−0.033) −0.037]	0.879 [(−0.039) −0.033]
CRP	0.655 [(−0.030) −0.048]	0.935 [(−0.050) −0.046]	0.607 [(−0.009) −0.015]	0.567 [(−0.008) −0.015]	0.004 (0.006–0.030)
ESR	0.152 [(−0.011) −0.068]	0.551 [(−0.034) −0.063]	0.001 (0.008–0.033)	0.039 (0.001–0.025)	0.856 [(−0.011) −0.014]

Correlation determined by multivariate regression analysis of analysis of variance (ANOVA) for 111 patients with rheumatoid arthritis (RA). Abbreviations are defined in Table 2 footnote. In the model, swollen joint count (SJC) is the dependent variable and sex, duration of symptoms, MCP-1, PTX3, CRP, and ESR are independent variables. Similarly, tender joint count (TJC), DAS 28, DAS28-MCP-1, and DAS28-CRP were analyzed individually as dependent variables in separate regression analyses. *P* values and confidence intervals (CIs) are shown.

### Evaluation of Subgroups by Stratified CRP and ESR Level

Mild inflammation was defined as a plasma CRP level <10 mg/L or an ESR <28 mm/hr. These cut-offs were selected based on the criteria of Pincus T et al [35]. Our hospital laboratory defines CRP<5 mg/L and ESR <15 mm/hr as normal.

Among RA patients with mild inflammation (i.e., serum CRP level <10 mg/L), duration of arthritic symptoms, but not sex, significantly correlated with all measures of arthritic activity (Table 4). Plasma MCP-1 significantly correlated with SJC, TJC, DAS28, and DAS28-CRP (Table 4). In contrast, blood PTX3, CRP, and ESR did not significantly correlate with any measure of clinical arthritic activity, with the exception of a correlation between ESR and DAS28-CRP (Table 4).

**Table 4:** Multivariate analysis of correlations between independent markers and measures of clinical arthritic activity for RA patients with serum CRP<10 mg/L.

	SJC	TJC	DAS28	DAS28-MCP1	DAS28-CRP
Sex	0.524 [(−4.481) −2.311]	0.141 [(−7.963) −1.168]	0.122 [(−2.129) −0.260]	0.507 [(−1.885) −0.944]	0.107 [(−2.150) −0.216]
Duration of symptoms	0.003 [(−0.058)–( −0.013)]	0.001 [(−0.082)–( −0.021)]	<0.001 [(−0.025)–( −0.009)]	0.040 [(−0.019)–( −0.000)]	<0.001 [(−0.024)–( −0.008)]
MCP-1	0.018 (0.001–0.015)	0.040 (0.000–0.019)	0.019 (0.000–0.005)	0.274 [(−0.001) −0.004]	0.032 (0.000–0.005)
PTX3	0.371 [(−0.205) −0.538]	0.346 [(−0.263) −0.735]	0.178 [(−0.042) −0.219]	0.182 [(−0.051) −0.259]	0.237 [(−0.052) −0.206]
CRP	0.344 [(−0.292) −0.822]	0.961 [(−0.767) −0.731]	0.200 [(−0.069) −0.323]	0.781 [(−0.200) −0.264]	0.127 [(−0.044) −0.344]
ESR	0.260 [(−0.083) −0.023]	0.209 [(−0.117) −0.026]	0.583 [(−0.014) −0.024]	0.690 [(−0.018) −0.027]	0.058 [(−0.036) −0.001]

Correlation was analyzed by estimating Pearson’s correlation coefficients for 55 patients with rheumatoid arthritis (RA); *P* values (CI) are shown. Abbreviations are defined in the footnotes for Tables 2 and 3.

Similarly, among patients with severe inflammation (CRP≥10 mg/L), duration of arthritic symptoms, but not sex, significantly correlated with all measures of arthritic activity (Table 5). The correlations of MCP-1 with SJC, DAS28, and DAS28-CRP were significant and stronger than the respective correlations with CRP (Table 5).

**Table 5:** Multivariate analysis of correlations between independent markers and measures of clinical arthritic activity for RA patients with serum CRP≥10 mg/L.

	SJC	TJC	DAS28	DAS28-MCP1	DAS28-CRP
Gender	0.456 [(−3.818) −1.739]	0.347 [(−4.719) −1.690]	0.129 [(−1.294) −0.169]	0.137 [(−1.397) −0.198]	0.184 [(−1.200) −0.236]
Duration of symptoms	0.006 [(−0.062)–( −0.011)]	0.010 [(−0.069)–( −0.010)]	0.010 [(−0.016)–( −0.002)]	0.003 [(−0.019)–( −0.004)]	0.005 [(−0.016)–( −0.003)]
MCP-1	0.056 (0.000–0.009)	0.185 [(−0.002) −0.009]	0.030 (0.000–0.003)	0.285 (0.000–0.002)	0.054 (0.000–0.002)
PTX3	0.259 [(−0.200) −0.055]	0.636 [(−0.182) −0.112]	0.785 [(−0.029) −0.038]	0.960 [(−0.036) −0.038]	0.897 [(−0.031) −0.035]
CRP	0.153 [(−0.093) −0.015]	0.077 [(−0.119) −0.006]	0.083 [(−0.027) −0.002]	0.392 [(−0.022) −0.009]	0.783 [(−0.016) −0.012]
ESR	0.029 (0.007–0.132)	0.045 (0.002–0.145)	<0.001 (0.015–0.048)	0.041 (0.001–0.037)	0.060 (0.000–0.031)

Correlation was analyzed by estimating Pearson’s correlation coefficients for 56 patients with rheumatoid arthritis (RA); *P* values (CI) are shown. Abbreviations are defined in the footnotes for Tables 2 and 3.

Among RA patients with mild inflammation (i.e., blood ESR <28 mm/hr), duration of arthritic symptoms, but not sex, significantly correlated with all measures of arthritic activity (Table 6). Plasma MCP-1 weakly correlated with SJC (Table 6). However, MCP-1, PTX3, CRP, and ESR were not correlated with any measure of arthritic activity (Table 6). The results were similar for RA patients with severe inflammation (blood ESR ≥28 mm/hr), except for the correlation between CRP and DAS28-CRP (Table 7).

**Table 6:** Multivariate analysis of correlations between independent markers and measures of clinical arthritic activity for RA patients with blood ESR <28 mm/hr.

	SJC	TJC	DAS28	DAS28-MCP1	DAS28-CRP
Sex	0.423 [(−4.716) −2.030]	0.113 [(−8.279) −0.921]	0.092 [(−2.156) −0.171]	0.366 [(−1.925) −0.732]	0.105 [(−2.064) −0.205]
Duration of symptoms	0.037 [(−0.056)–( −0.002)]	0.037 [(−0.085)–( −0.011)]	0.007 [(−0.023)–( −0.004)]	0.027 [(−0.023)–( −0.001)]	0.006 [(−0.022)–( −0.004)]
MCP-1	0.060 (0.000–0.016)	0.391 [(−0.006) −0.016]	0.151 (0.000–0.005)	0.995 [(−0.003) −0.003]	0.192 (0.000–0.005)
PTX3	0.940 [(−1.261) −1.170]	0.687 [(−1.314) −2.002]	0.738 [(−0.350) −0.488]	0.226 [(−0.189) −0.768]	0.705 [(−0.332) −0.485]
CRP	0.270 [(−0.066) −0.279]	0.817 [(−0.208) −0.262]	0.586 [(−0.043) −0.075]	0.567 [(−0.087) −0.049]	0.188 [(−0.020) −0.096]
ESR	0.219 [(−0.345) −0.083]	0.798 [(−0.255) −0.329]	0.306 [(−0.036) −0.111]	0.234 [(−0.034) −0.134]	0.674 [(−0.087) −0.057]

Correlation was analyzed by estimating Pearson’s correlation coefficients for 37 patients with rheumatoid arthritis (RA); *P* values (CI) are shown. Abbreviations are defined in the footnotes for Tables 2 and 3.

**Table 7:** Multivariate analysis of correlations between independent markers and measures of clinical arthritic activity for RA patients with blood ESR ≥28 mm/hr.

	SJC	TJC	DAS28	DAS28-MCP1	DAS28-CRP
Sex	0.560 [(−3.051) −1.913]	0.413 [(−4.558) −1.894]	0.279 [(−1.226) −0.359]	0.248 [(−0.349) −1.325]	0.312 [(−1.265) −0.410]
Duration of symptoms	0.001 [(−0.058)–( −0.015)]	0.002 [(−0.069)–( −0.017)]	0.001 [(−0.018)–( −0.005)]	0.002 [(−0.017)–( −0.004)]	0.001 [(−0.018)–( −0.005)]
MCP-1	0.077 (0.000–0.009)	0.208 [(−0.002) −0.009]	0.123 (0.000–0.003)	0.223 (0.000–0.002)	0.161 (0.000–0.003)
PTX3	0.238 [(−0.198) −0.050]	0.554 [(−0.192) −0.104]	0.955 [(−0.037) −0.035]	0.507 [(−0.051) −0.026]	0.800 [(−0.043) −0.033]
CRP	0.972 [(−0.044) −0.046]	0.698 [(−0.064) −0.043]	0.290 [(−0.006) −0.020]	0.199 [(−0.005) −0.023]	0.009 [(−0.005) −0.033]
ESR	0.181 [(−0.019) −0.097]	0.227 [(−0.027) −0.111]	0.267 [(−0.007) −0.026]	0.474 [(−0.011) −0.024]	0.744 [(−0.021) −0.015]

Correlation was analyzed by estimating Pearson’s correlation coefficients for 74 patients with rheumatoid arthritis (RA); *P* values (CI) are shown. Abbreviations are defined in the footnotes for Tables 2 and 3.

In summary, when RA patients were divided into two sub-groups (serum CRP levels <10 or ≥10 mg/L), plasma MCP-1 correlated better with indicators of arthritic activity (SJC, TJC, DAS28 and DAS28-CRP) than did CRP (Tables 4 and 5).

### Correlation between Intracellular PTX3 and MCP-1 Staining in Leukocytes and Plasma PTX3, MCP-1, and Indicators of RA Disease Activity

Specimens from 8 RA patients underwent cell surface and intracellular staining at the time of diagnosis. Plasma MCP-1 levels were positively correlated with intracellular MCP-1 levels of CD62L^+^-activated monocytes (rho  = 0.841 and *P*  = 0.036). TJC also positively correlated with intracellular MCP-1 levels of CD11c^+^CD66b^+^-activated PMNs (rho  = 0.812 and *P*  = 0.050). These results suggest that MCP-1^+^-activated phagocytes accurately reflect plasma MCP-1 levels and TJC. Conversely, plasma MCP-1 was negatively correlated with intracellular PTX3 levels of CD11c^+^CD66b^+^-activated PMNs (rho = −1.000 and *P*<0.001). Similarly, plasma MCP-1 level was inversely correlated with intracellular PTX3 levels of CD11b^+^CD62L^+^-activated monocytes (rho = −1.000 and *P*<0.001). This negative association between MCP-1 and PTX3 was similar to those between plasma PTX3 and intracellular MCP-1 levels of CD11b^+^-activated monocytes (rho = −1.000 and *P*<0.001), between intracellular MCP-1 levels and intracellular PTX3 levels of CD19^+^CD23^+^- activated B cells (rho = −0.900 and *P*  = 0.037), and between plasma MCP-1 and intracellular PTX3 levels of CD3^+^CD69^+^-activated T cells (rho = −0.900 and *P*  = 0.037). In sum, these results suggest that MCP-1 (pro-inflammatory) and PTX3 might have opposing roles in RA inflammation.

## Discussion

Serum CRP and blood ESR levels have long been routinely used to monitor clinical arthritic activity in RA patients for many years [36]–[38]. It is uncertain whether other biomarkers [5], [15] are useful in monitoring RA patients [18], [22], [23].

We found that plasma MCP-1 (Tables 2 and 3)–particularly, the adapted DAS28-MCP-1 (Table 2 and Fig. 1A–1F)–was useful in evaluating RA disease activity. Our results are compatible with those of a previous study of 36 RA patients that showed a significant correlation between plasma MCP-1 levels and SJC [22]; further, ours had a further extension on the positive correlation between MCP-1 levels and DAS28 (Table 2). However, as compared with our findings, the results of that study differed with regard to the correlation of ESR/CRP with SJC (Table 2) [22]. Our results also differed from the findings of a study of 47 RA patients [23], which found a significant correlation between serum MCP-1 levels and ESR/CRP levels (the association between MCP-1 and ESR in our study was rho  = 0.197 and *P*  = 0.038; that between MCP-1 and CRP was rho  = 0.125 and *P*  = 0.189) and which found no correlation between serum MCP-1 levels and SJC or DAS28 (Table 2). We further found that the adapted DAS28-MCP-1 strongly correlated with DAS28 during the 6-month observation period (Figure 1). However, the results of the previous study differed with regard to the correlation between MCP-1, CRP, or ESR and SJC or DAS28 (Table 2). It should be noted that those two previous reports did not include newly diagnosed RA patients [22], [23]. Thus, the differences between previous and present findings could be due to our inclusion of both newly diagnosed DMARD-untreated RA patients (43.2%) and regularly DMARD-treated RA patients (56.8%) as well, to the sample size of our study (Tables 1, 2, and 3; ref. 22, 23). Moreover, the modest cross-sectional correlations of MCP-1 with SJC, TJC, and DAS28 (Table 2), the strong longitudinal correlations between DAS-MCP-1 and DAS28 during the 6-month period (Figure 1), and the strong cross-sectional correlation between DAS28-MCP-1 and DAS28-CRP suggest that MCP-1 could be used as a biomarker for RA patients in the future. In contrast, the results for PTX3–once regarded as a promising biomarker for RA disease activity [18]–were disappointing (Tables 2 and 3).

It is unclear if PTX3 has a protective role, considering the inverse relationship of plasma MCP-1 and PTX3 levels with intracellular expressions of PTX3 and MCP-1 in activated cells, respectively. However, the possibility of such a role is strengthened by the fact that PTX3 secretion from RA monocytes was induced by an immunosuppressant cytokine (IL-10), but not by pro-inflammatory cytokines (IFN-γ and IL-1β not shown; the effect of IFN-γ on PTX3 production was the same as that described previously in human peripheral blood mononuclear cells in ref. [3]). The effects of IL-10 and IFN-γ on PTX3 production were similar to those reported previously [39] . However, in the synovial tissue, PTX3 possibly acts by amplifying complement-mediated inflammation and subsequent damage [39] . Hence, present evidences suggest that PTX3 is like a two-edged sword in protecting against autoimmunity and favoring autoimmunity [39]. In particular, PTX3 polymorphism might explain opposite functions in diverse settings. Since the definite role of PTX3 in rheumatoid arthritis is uncertain, the question of whether PTX3 suppresses or promotes RA inflammation is worthy of a future case-control association study [40], [41].

There is insufficient evidence to determine whether MCP-1 or DAS28-MCP-1 monitoring (Figure 1, Tables 2 and 3) is useful in monitoring disease progression and avoiding joint destruction (radiological progression), as good as previously described for serum CRP levels [42], [43]. In particular, whether DAS28-MCP-1 scores are useful in assessing RA patients with a low initial ESR levels is unknown [44] although the MCP-1 level was a better indicator of arthritic activity in RA patients with low CRP levels (Table 4). Moreover, a previous study somewhat surprisingly showed no favorable immunohistologic or clinical effects among RA patients treated with a human monoclonal anti–CCL2/MCP-1 [21]. Nevertheless, the correlation of plasma MCP-1 with tender and swollen joint count was better than the poor correlations of ESR and CRP with measures of clinical disease activity in RA (Tables 2 and 3) [45].

The main weakness of this study was that that RA patients were recruited consecutively, rather than a full cohort (only 48 newly diagnosed patients were followed up as a cohort, for 6 months; Figure 1). Most previous cohort studies enrolled a relatively homogeneous group of RA patients with ≥6 active swollen joints and followed them for a finite time period after administration of a trial medication [46]–[48]. The current study (which includes newly diagnosed DMARD-untreated and regularly treated RA patients) may not be sufficient with regard to variation in arthritis severity and follow-up period. These weaknesses can be addressed by studying MCP-1 and DAS28-MCP-1 in an observational cohort (rather than a drug trial cohort).

Another weakness of our study was that the relationship between very low plasma CRP levels (<3.00 mg/L) and clinical arthritic activity could not be adequately investigated [49]. Previous studies found that the association between high-sensitivity CRP and RA disease activity was stronger than the association between ESR and RA disease activity [49], but this was not the case in the present study (Tables 2 and 3). This discrepancy is probably due to exclusion of recently enrolled (<6 months) RA patients and the fact only 12% of RA patients (versus 27% in our study) had serum CRP levels >23 mg/L in the previous report [49]. Moreover, CRP could not be precisely determined in 12 of our patients, ie, those with a CRP level of <3.00 mg/L. Whether or not this phenomenon is due to medication effects cannot be determined [49]. Nevertheless, the combination of newly diagnosed and regularly treated RA patients in this study (Tables 2 and 3) accurately represents current clinical reality.

Thirdly, the RF positive rate at 59% (similar for anti-CCP antibody) was low in our RA population (Table 1). In our opinion, the time period between RF/anti-CCP antibody examination and the beginning of arthritic symptoms was probably critical. In our study, 48 newly diagnosed RA patients had 50% of positive plasma anti-CCP antibody, in contrast to 70% in 63 regularly treated RA patients (similar for positive RF rates). The duration of arthritic symptoms of the latter (47.5 months [18.0, 96.0] expressed as median [25^th^ percentile, 75^th^ percentile]) was significantly longer than that of the former (6.0 months [2.6, 23.4]) (*P*<0.001 by Mann-Whitney U test). Moreover, 31 and 44 RA patients from two respective recent studies in Taiwan [50], [51] and 122 RA patients from eastern France [52] had >75% of RF positivity (ref. 51 also reported a high rate of positive anti-CCP antibodies >95%) and included most of RA patients having a disease duration >1 year (ref. 51, personal communication). In contrast, three other studies published from UK, Sweden, and Japan [53]–[55] had low rates of positive RF (49%, 66%, or 50%, respectively) and low rates of positive anti-CCP antibodies (52%, 65%, or 32%, respectively); all RA patients had a disease duration <1 year (a 3-month duration of disease for the UK study, ref. 53). Hence, it appears that RA patients with a shorter disease duration are less likely to be RF-positive and anti-CCP-positive, though the timing of RF and anti-CCP antibody examination and the boundary between short and long disease duration of our patients were not necessarily similar to those RA patients published previously [51]–[55]. It should be noted that 33.3% of our regularly treated RA patients had RF re-checked on enrollment due to loss of the data and anti-CCP antibodies were measured on enrollment for all RA patients (Table 1 footnote). Moreover, whether this finding is related to medication effects is unanswered yet. Nevertheless, a well-designed prospective study with a large sample size is needed to address this interesting finding.

Fourthly, whether or not DAS28-MCP-1 can be effectively used in monitoring disease progression in a large diverse population of RA patients without and after DMARD treatment was not completely answered in this study, although we did show that DAS28-MCP-1 scores correlated well with DAS28 scores during a 6-month period in 48 newly diagnosed RA patients (Figures 1C–1F). Lastly, DAS28-MCP-1 has not been validated by radiographic studies and assessment of physical function; hence, a well-planned study is needed to verify the use of DAS28-MCP-1 against DAS28 (with inclusion of ESR) in RA patients [56]. Furthermore, it is necessary to evaluate criterion validity and construct validity to address discrepancies in using DAS28-MCP-1, DAS, and DAS-CRP to categorize RA patients [57].

In conclusion, in contrast to PTX3, plasma MCP-1 is one of the best indicators of clinical arthritic activity in RA patients in terms of SJC, TJC, DAS28, DAS28-CRP, and DAS28-MCP-1. Data on the usefulness of MCP-1 for different ranges of CRP and ESR is preliminary and requires further verification. DAS28-MCP-1 correlated well longitudinally with DAS28 (Figure 1), and DAS28-MCP-1 and DAS28-CRP were strongly correlated. These findings indicate that adapted DAS28-MCP-1 may be useful in evaluating RA disease activity. However, this observation must be interpreted with caution in view of the limited sample size and short duration of follow-up in the current study. Nevertheless, as compared with ESR and CRP, MCP-1 had a stronger individual correlation (higher correlation coefficients and lower *P*-values) with swollen joint count and tender joint count (Tables 2 and 3). In particular, a new biomarker for RA inflammation is very much needed for clinical trials and daily clinical practices, as patients with active RA can have normal inflammatory markers (ESR/CRP) and vice versa [58], [59]. Moreover, ESR and CRP levels represent different underlying pathophysiologies and the DAS28-CRP threshold values differ from those of the DAS28-(ESR) [60]. Similarly, MCP-1 has a different cell source of production and a different underlying pathophysiology from ESR and CRP (see Introduction). Therefore, use of plasma MCP-1 and DAS28-MCP-1 in clinical RA practice is promising, pending validation by future studies. Namely, a much larger RA patient population is needed in order to calculate DAS thresholds that discriminate active disease from inactive disease and remission from non-remission. Confirmation by studies of different racial and ethnic groups is also desirable [26].
